# Laboratory Evaluation of *Beauveria bassiana* for Biological Control of the Elm Leaf Beetle, *Pyrrhalta aenescens* (Coleoptera: Chrysomelidae)

**DOI:** 10.3390/insects17060626

**Published:** 2026-06-14

**Authors:** Binglin Wang, Ziqun Guo, Wanying Shang, Liyuan Yang

**Affiliations:** College of Forestry, Shenyang Agricultural University, Shenyang 110866, China; wbl0803@126.com (B.W.); guoziqun0376@163.com (Z.G.); swy20030712@163.com (W.S.)

**Keywords:** *Beauveria bassiana*, *Pyrrhalta aenescens*, entomopathogenic fungus, immune physiology, virulence bioassay, biological control

## Abstract

To identify novel field control strategies against *Pyrrhalta aenescens* (Coleoptera: Chrysomelidae) and provide scientific support for its biocontrol in urban tree management, this study investigated the virulence of *Beauveria bassiana* against this pest under laboratory conditions, as well as its physiological and biochemical effects. Bioassays using the dipping method showed that *B. bassiana* was pathogenic to all developmental stages of *P. aenescens*, with the highest virulence observed against early-instar larvae (1st and 2nd instars). For these stages, corrected mortality and mycosis rate were positively correlated with conidial concentration, and the median lethal time (LT_50_) was the shortest. In contrast, pupae and eggs exhibited the strongest resistance to fungal infection. In leaf-disk choice tests, larvae significantly preferred untreated leaves or those treated with low concentrations of *B. bassiana*, displaying a concentration-dependent repellent response to the fungus. Physiological measurements revealed that larval body length and weight gain were significantly inhibited following fungal exposure. Further analysis indicated that *B. bassiana* infection markedly reduced total hemocyte counts and triggered intense melanization and nodulation responses, particularly in younger larvae. Overall, these results suggest that *B. bassiana* has strong potential for the biological control of *P. aenescens*. Control measures targeting early-instar larvae are recommended for cost-effective management, providing a scientific basis for developing eco-friendly control technologies based on this entomopathogenic fungus.

## Simple Summary

This paper presents a laboratory study aimed at evaluating the efficacy of *Beauveria bassiana* against the elm leaf beetle, *Pyrrhalta aenescens*. The pathogenicity of *B. bassiana* spore suspensions against eggs, larvae of all instars, pupae, and adults of *P. aenescens* was determined using a dipping method. A leaf-disk choice assay revealed that the larvae significantly avoided leaf disks treated with *B. bassiana*, and this avoidance behavior was positively correlated with the spore concentration. The study further investigated the physiological and immunological impacts of *B. bassiana* infection on the host. Data indicated that the fungal infection significantly inhibited larval growth in terms of body length and weight gain. Regarding immune responses, the infection led to a significant decrease in the total hemocyte count and triggered intense melanization and nodulation reactions, with immune responses being more pronounced in early-instar larvae. This study confirmed that *B. bassiana* has significant direct lethal and various sublethal effects on *P. aenescens*, with the most pronounced efficacy observed against early-instar larvae. *Beauveria bassiana* demonstrates considerable application potential in the biological control of *P. aenescens*, providing a scientific basis for developing environmentally friendly control technologies based on this fungus.

## 1. Introduction

The elm leaf beetle, *Pyrrhalta aenescens* Fairmaire (Coleoptera: Chrysomelidae), is a major defoliating pest of elm trees (*Ulmus* spp.) in China. Both adults and larvae feed on the foliage; under severe infestation, defoliation rates can exceed 95%, severely impairing tree growth and threatening the ecological stability of urban landscapes [1,2,3]. This species is widely distributed across Northeast, North, and Northwest China, with two generations per year in Liaoning Province, where it poses a high outbreak risk [4,5]. Current management practices rely heavily on chemical insecticides, despite the availability of silvicultural, physical, and biological control measures [6,7]. Prolonged chemical use has led to pest resurgence, secondary pest outbreaks, and environmental contamination, underscoring the urgent need for sustainable, environmentally friendly control strategies [8].

Entomopathogenic fungi offer a promising alternative due to their broad host range, high target specificity, and low risk of resistance development [9,10,11]. Among these, *Beauveria bassiana* (Bals.–Criv.) Vuill. Is one of the most widely studied and commercialized fungal biocontrol agents, with documented infections in over 700 insect species across 10 orders while remaining safe for humans and non-target organisms [12,13]. Its efficacy against coleopteran pests is well documented, including against species such as *Basilepta melanopus*, *Monochamus alternatus*, and scarab larvae [14,15,16]. International studies have further demonstrated the potential of *B. bassiana* against chrysomelid pests, including *Diabrotica virgifera* and *Oulema melanopus* [17,18]. Despite this body of evidence, no study to date has evaluated the pathogenicity of *B. bassiana* against *P. aenescens*, representing a significant knowledge gap.

Beyond direct mortality, entomopathogenic fungi can induce a range of sublethal effects on host insects, including alterations in feeding behavior, growth, and immune physiology. *B. bassiana* infection has been shown to suppress feeding and weight gain in *Ootheca mutabilis* and *Spodoptera exigua* [19,20], whereas endophytic colonization can deter feeding in stem-boring pests [21,22]. Conversely, some studies suggest fungi may manipulate host feeding to enhance their own development, as observed in *Drosophila melanogaster* [23]. With respect to immune responses, fungal infection typically triggers cellular defenses such as hemocyte proliferation and nodule formation. *B. bassiana* significantly reduces total hemocyte counts in *Bombyx mori* [24], while infection with *Metarhizium anisopliae* causes dynamic changes in hemocyte counts in *Algedonia coclesalis* [25]. Furthermore, infection with *B. bassiana* leads to a decrease in the total hemocyte numbers in the insect hosts [26]. Nodule formation, a key indicator of insect immunocompetence, is also influenced by fungal infection [27,28,29]. These physiological and immune responses are critical for understanding the full impact of a biocontrol agent and for predicting its performance under field conditions.

Given the absence of information on the interaction between *B. bassiana* and *P. aenescens*, this study aimed to: (1) evaluate the virulence of a *B. bassiana* strain against *P. aenescens* under controlled laboratory conditions; (2) assess the effects of *B. bassiana* infection on body length, body weight, settling behavior, total hemocyte counts, and nodule formation; and (3) provide a preliminary characterization of the pathogenic mechanisms involved. The findings will support the selection of effective *B. bassiana* strains for future field applications and contribute to the development of integrated, environmentally sustainable management strategies for elm leaf beetles in urban landscapes.

## 2. Materials and Methods

### 2.1. Fungal Strain, Culture Conditions, and Experimental Materials

Fungal strain and identification. The *B. bassiana* strain was isolated from mummified adults of *P. aenescens* (Shenyang Agricultural University, Shenyang, China) and deposited at (CGMCC) (No. SYAUBB001). Morphological identification was performed on 15-day PDA cultures by observing colony characteristics and conidial structures under light microscopy. Molecular identification followed standard protocols: genomic DNA was extracted via the CTAB method; the ITS (primers ITS1/ITS4) region was amplified by PCR (95 °C 5 min; 35 cycles of 94 °C 30 s, 55–58 °C 30 s, 72 °C 1 min; 72 °C 10 min) and sequenced bidirectionally. Sequences were BLASTn-compared (https://blast.ncbi.nlm.nih.gov/Blast.cgi, accessed on 11 October 2024) against GenBank. Phylogenetic analysis was conducted in MEGA 11.

Culture conditions. The fungus was maintained on homemade potato dextrose agar (PDA) prepared from 200 g potato, 20 g of D-(+)-glucose (Shanghai Aladdin Biochemical Technology Co., Ltd., Shanghai, China, CAS: 50-99-7), 20 g of agar (Shanghai Aladdin Biochemical Technology Co., Ltd., Shanghai, China; ash ≤ 1.5%, gel strength 700–900 g/cm^2^), and distilled water to 1 L.

Host plants and insects. *U. pumila* seedlings (40–50 cm height, 1-year-old) were cultivated indoors in plastic pots (30.5 cm in diameter × 20 cm in height) filled with commercial potting soil (purchased from a local nursery) under controlled conditions: temperature 25 ± 2 °C, relative humidity 60 ± 5%, photoperiod 16 L:8 D, and light intensity 8000–10,000 lux. Plants were watered every 5 days without any fertilizer application. *P. aenescens* individuals were originally collected from the Botanical Garden of Shenyang Agricultural University and reared in cages (60 cm × 60 cm × 90 cm) on fresh elm leaves. Second- to third-instar larvae from heavily infested stands were used to establish the laboratory colony. After three generations of indoor rearing, individuals exhibiting strong locomotor activity, uniform development, and consistent physiological status were selected for bioassays.

Data analysis. Statistical analyses were performed using IBM SPSS Statistics 27 (IBM Corp., Armonk, NY, USA) and Microsoft Excel 2016 (Microsoft Corp., Redmond, WA, USA).

### 2.2. Virulence Bioassay of B. bassiana Against Different Life Stages of P. aenescens

Virulence was evaluated using a dipping method as described by Inglis et al. [30]. Spores were harvested from 15-day-old PDA cultures by gently rinsing the colony surface with sterile distilled water containing 0.1% (*v*/*v*) Tween-80. The resulting suspension was vortexed for 30 s to dislodge conidia, then filtered through four layers of sterile cheesecloth to remove mycelial fragments. Spore viability was assessed prior to bioassays by mixing 100 μL of the suspension with an equal volume of 0.05% (*w*/*v*) Methyl blue solution (Shanghai yuanye Bio-Technology Co., Ltd., Shanghai, China, LOT: KR37662A) on a microscope slide; non-viable conidia stained blue, whereas viable conidia remained unstained. Only suspensions with >90% viability were used. Spore concentrations were adjusted to 1.0 × 10^6^, 1.0 × 10^7^, and 1.0 × 10^8^ spores/mL using a hemocytometer, with each concentration verified by three independent counts.

Egg masses (15 masses/group), 1st- to 3rd-instar larvae (15 larvae/group), pupae (15 pupae/group), and adults (15 adults/group) were individually dipped in spore suspensions for 5 s. Control groups were treated with sterile distilled water containing 0.1% Tween-80. After treatment, excess liquid was removed with sterile filter paper, and the insects were transferred to plastic rearing containers (10.8 × 10.8 × 7.5 cm) lined with moistened sterile filter paper. Containers were maintained in a climatic chamber at 25 ± 1 °C, 75 ± 5% relative humidity, and a photoperiod of 14:10 h (L:D), with fresh sterile elm leaves provided daily. Each treatment consisted of three replicates, yielding a total of 45 individuals per developmental stage-concentration combination (*n* = 45).

Mortality was recorded daily. Dead individuals were removed daily, surface-sterilized by dipping in 75% ethanol for 30 s followed by three rinses in sterile distilled water, and transferred individually to sterile Petri dishes lined with moistened filter paper. Cadavers were incubated in a climatic chamber at 25 ± 1 °C and >95% relative humidity for 7 days to promote fungal sporulation. Mycosis was confirmed by the emergence of characteristic white mycelium and conidia on the cadaver surface, with the fungal identity verified by microscopic examination (400× magnification) of conidial morphology consistent with *B. bassiana* (ovoid conidia, 2–3 × 1.5–2 μm). Individuals showing no fungal growth after 7 days were classified as non-mycosis-related mortality. Corrected mortality was calculated on day 5, the mycosis rate on day 7, and the median lethal time (LT_50_) was estimated using probit analysis according to Finney (1971) [31], as implemented in IBM SPSS Statistics 27 (IBM Corp., Armonk, NY, USA). Concentration-mortality data were subjected to probit regression to generate lethal time estimates with associated 95% confidence intervals.

(1) $$\mathrm{Mortality}\;(\%)=\frac{\mathrm{Number}\;\mathrm{of}\;\mathrm{dead}\;\mathrm{larvae}}{\mathrm{Total}\;\mathrm{number}\;\mathrm{of}\;\mathrm{larvae}}\times 100\%$$

(2) $$\mathrm{Corrected}\;\mathrm{mortality}\;(\%)=\frac{{\mathrm{Mortality}}_{\mathrm{Treatment}}-{\mathrm{Mortality}}_{\mathrm{control}}}{1-{\mathrm{Mortality}}_{\mathrm{control}}}\times 100\%$$

(3) $$\mathrm{Mummification}\;\mathrm{rate}\;(\%)=\frac{\mathrm{Number}\;\mathrm{of}\;\mathrm{mummified}\;\mathrm{larvae}}{\mathrm{Total}\;\mathrm{number}\;\mathrm{of}\;\mathrm{larvae}}\times 100\%$$

### 2.3. Settling Preference Assay

**Figure 1:**
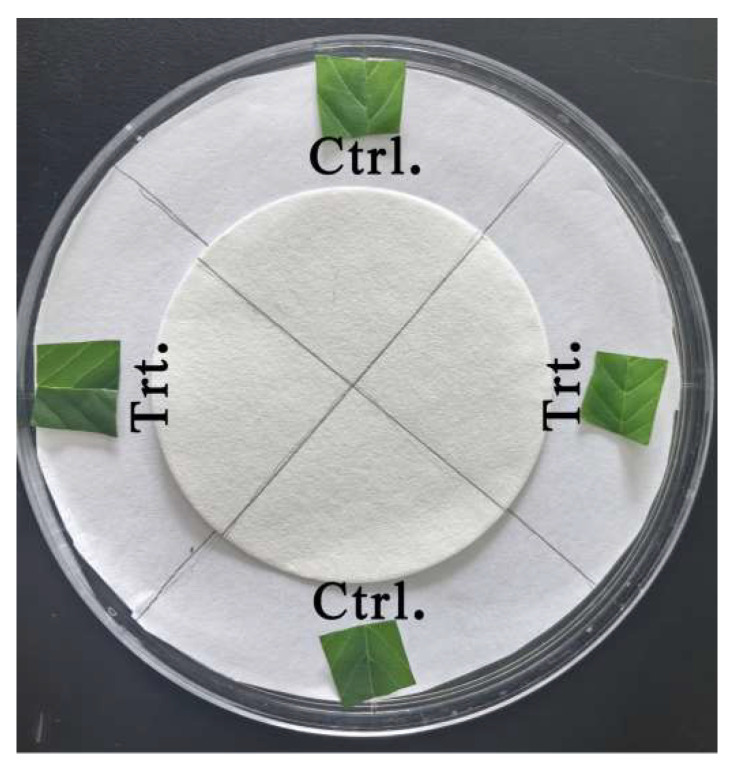
Schematic diagram of elm leaf disks.

Settling preference was assessed using a leaf disk choice test. Healthy *U. pumila* leaf disks (2 cm diameter) were immersed in *B. bassiana* spore suspensions (1.0 × 10^6^, 1.0 × 10^7^, or 1.0 × 10^8^ spores/mL) or sterile distilled water (control) for 30 min, then air-dried for 30 min under sterile conditions. Two leaf disks from each group were placed symmetrically in a Petri dish (9 cm diameter, Figure 1). Third-instar larvae were selected for this assay because they exhibit robust feeding activity and are large enough to permit accurate observation, while still being representative of the larval feeding stage [32]. Ten third-instar larvae, starved for 24 h, were introduced into each dish. After 20 h, the number of larvae settled on each leaf disk was recorded. Settling preference was determined by comparing larval distribution among groups. This assay reflects behavioral avoidance/selection rather than direct feeding damage, as actual leaf consumption was not quantified. Each treatment was replicated three times.

### 2.4. Effects of B. bassiana on Growth and Development of P. aenescens

*Ulmus pumila* seedlings (50 cm in height) were inoculated with *B. bassiana* spore suspensions (1.0 × 10^6^ spores/mL) using a root-drenching method (500 mL per seedling, applied three times at 5-day intervals). Control seedlings received an equal volume of sterile distilled water. This method was employed to evaluate the systemic uptake and potential endophytic colonization of the fungus within the plant tissues.

After the third root-drenching treatment, leaf tissues from treated and control seedlings were excised, surface-sterilized (75% ethanol, 30 s; followed by three rinses in sterile distilled water), and plated on PDA to verify endophytic colonization of *B. bassiana*. Thirty first-instar larvae with uniform development were then placed directly onto the foliage of each seedling and reared in mesh cages (60 cm × 60 cm × 90 cm) at 25 ± 2 °C under ambient laboratory conditions. Each treatment comprised three replicates (three seedlings).

Larval body length and weight were recorded daily at the end of each instar. Body length was measured to the nearest 0.1 mm using digital vernier calipers (*n* = 20 larvae per treatment per instar, selected from surviving larvae), and body weight was determined to the nearest 0.1 mg using an analytical balance (*n* = 20 larvae per treatment per instar). The developmental duration and survival rate were also monitored.

### 2.5. Hemocyte Count and Melanotic Nodule Formation

Hemocyte count. Third-instar larvae were selected for immunological assays because their larger body size facilitates hemolymph collection and dissection, and their immune system is fully developed yet still responsive to fungal challenge, providing a reliable model for cellular defense assessment [33]. Third-instar larvae were inoculated with *B. bassiana* using the dipping method and sampled at 0, 6, 12, 24, 36, and 48 h post-inoculation. Hemolymph was collected by making a small incision in the anterior region of the larva with a sterile dissecting needle. Exuding hemolymph (10 μL) was immediately mixed with hemocyte dilution fluid (2% acetic acid with 1% methylene blue, 100:1 *v*/*v*), loaded onto a hemocytometer, and examined under a light microscope at 400× magnification. Total hemocyte counts were determined from three independent replicates per time point.

Melanotic nodule observation. Larvae were treated as described above and sampled at the same time intervals. Insects were anesthetized on ice for 30 min and then dissected longitudinally from the posterior to the anterior abdomen using dissecting scissors. The body cavity was pinned open on a black wax dissecting dish and gently rinsed with phosphate-buffered saline (PBS, 0.01 M, pH 7.4) (Shanghai yuanye Bio-Technology Co., Ltd., Shanghai, China, LOT: KS364630) to remove hemolymph and contaminants, and the number of melanotic nodules on the digestive tract surface was counted under a stereomicroscope. Each treatment consisted of three replicates.

## 3. Results

### 3.1. Laboratory Virulence of B. bassiana Against P. aenescens

Treatment of different developmental stages of *P. aenescens* with *B. bassiana* spore suspensions resulted in distinct pathological symptoms and mortality patterns.

**Figure 2:**
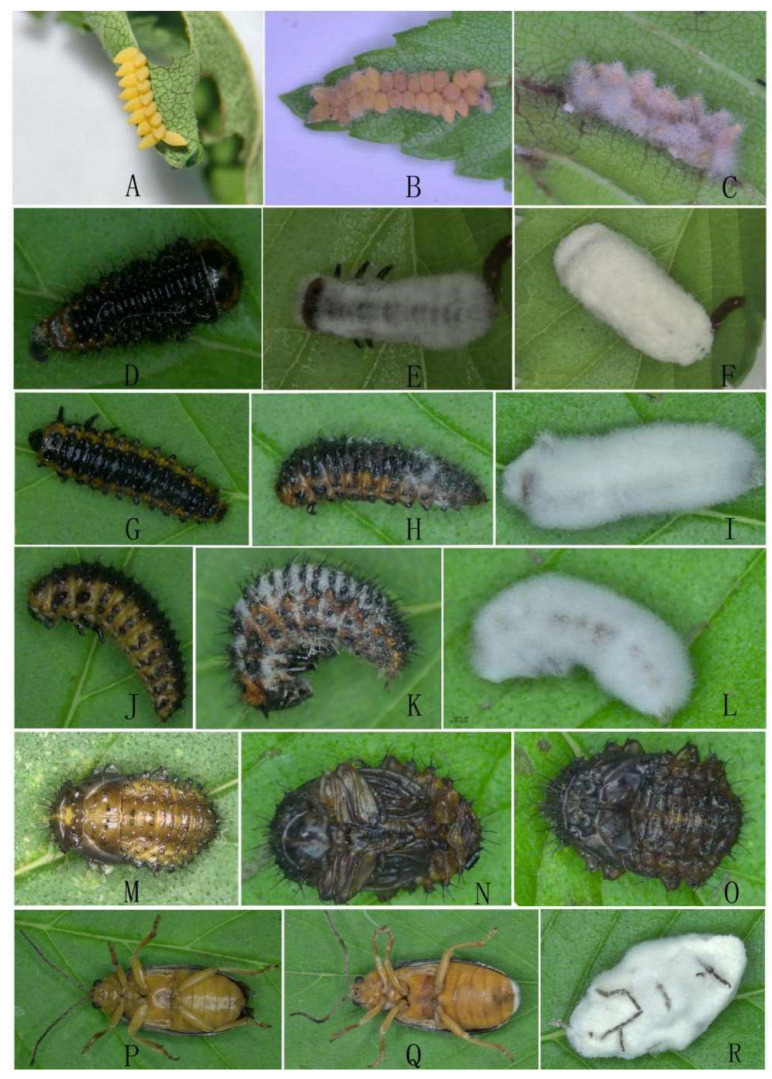
Mycelial growth on *P. aenescens* after *B. bassiana* treatment: (**A**–**C**) Eggs; (**D**–**F**) 1st–instar larvae; (**G**–**I**) 2nd–instar larvae; (**J**–**L**) 3rd–instar larvae; (**M**–**O**) pupae; (**P**–**R**) adults.

*Egg stage*. Mortality was observed 3–4 days after treatment, characterized by the progressive darkening of the chorion and the emergence of sparse white mycelia on the egg surface. As fungal colonization advanced, the eggs underwent gradual desiccation and shriveling, culminating in complete mycelial coverage of the egg mass (Figure 2A–C).

*Larval stages*. Age-dependent differences in susceptibility were evident, with mortality initiating at 1–2 days (1st instar), 3–4 days (2nd instar), and 4–5 days (3rd instar) after treatment. Infected larvae exhibited a characteristic disease progression: abdominal discoloration from bright yellow to pink–purple, followed by loss of motility, body rigidity, surface mycelial growth, and complete mummification (Figure 2D–L).

*Pupal stage*. Mortality was observed 6–7 days after treatment, characterized by progressive cuticular darkening and sparse superficial myceliation (Figure 2M–O).

*Adult stage*. Sublethal effects manifested 1–2 days after treatment, including reduced locomotory activity and feeding cessation. Mortality occurred 3–4 days, with a concomitant loss of cuticular luster. Post-mortem mycelial emergence from the integument culminated in complete mycelial mummification (Figure 2P–R).

Virulence assays revealed significant stage-dependent variation in susceptibility to *B. bassiana* (Table 1). A clear dose-response relationship was observed across all stages.

First-instar larvae were the most susceptible, with 73.33% corrected mortality and 100% mycosis at 1.0 × 10^8^ spores/mL, and the shortest LT_50_ of 3.470 days. Second-instar larvae showed intermediate susceptibility (33.33% mortality, 95.53% mycosis, LT_50_ 4.396 days), whereas third-instar larvae were more refractory (11.13% mortality, 97.13% mycosis, LT_50_ 6.028 days).

Eggs displayed moderate sensitivity (corrected mortality: 28.87–57.80%; mycosis: 44.47–73.33%; LT_50_: 5.158–7.092 days). Pupae were the most refractory stage, with minimal mortality (0–4.47%) and mycosis (2.20–40.00%), and LT_50_ exceeding 19 days at the lowest concentration. Adults showed delayed but complete susceptibility (100% mycosis by day 7; LT_50_: 5.018–5.392 days).

Susceptibility rankings converged: day-5 mortality followed pupa < third instar < second instar < adult < egg < first instar; LT_50_ followed the inverse sequence. These patterns identify early-instar larvae as the optimal intervention target and pupal/egg stages as the most recalcitrant.

Pathogenicity parameters exhibited significant positive correlations with spore concentration across all developmental stages. Susceptibility rankings varied by metric: day-5 mortality followed the order pupa < third instar < second instar < adult < egg < first instar; day-7 mycosis followed pupa < third instar < egg < second instar < first instar < adult; and LT_50_ followed the inverse sequence first instar < second instar < adult < third instar < egg < pupa. These convergent patterns indicate that pupal and egg stages represent the most refractory targets for *B. bassiana*-mediated control, followed by third-instar larvae. In contrast, first-instar and second instars showed the highest susceptibility with a rapid onset of visible mycosis.

### 3.2. Settling Preference P. aenescens in Response to B. bassiana

Settling preference tests using third-instar larvae and the leaf disk method revealed concentration-dependent avoidance behavior toward *B. bassiana*-treated foliage (Figure 2).

*Comparisons with control.* At 1.0 × 10^6^ spores/mL, no significant preference was observed during the initial 4 h; thereafter, the larvae exhibited a significant shift toward the control disks, with 2–3 individuals per group (27 ± 6%) showing no selection. At 1.0 × 10^7^ spores/mL, similar patterns emerged, with no preference before 4 h, followed by significant avoidance of the treated disks from 4 to 16 h, and 2–3 non-responders per group (23 ± 6%). At 1.0 × 10^8^ spores/mL, immediate and significant avoidance was observed throughout the assay, with only 1–2 individuals per group (17 ± 6%) failing to discriminate.

*Between-treatment comparisons*. When offered paired treated disks, the larvae discriminated significantly between concentrations. At 12 h, they preferred the 1.0 × 10^6^ over the 1.0 × 10^7^ spores/mL, and strongly preferred the 1.0 × 10^6^ over 1.0 × 10^8^ spores/mL. Notably, no significant preference was detected between the 1.0 × 10^7^ and 1.0 × 10^8^ spores/mL treatments, with 4–5 individuals per group (47 ± 6%) showing no selection—indicating threshold effects at higher concentrations.

**Figure 3:**
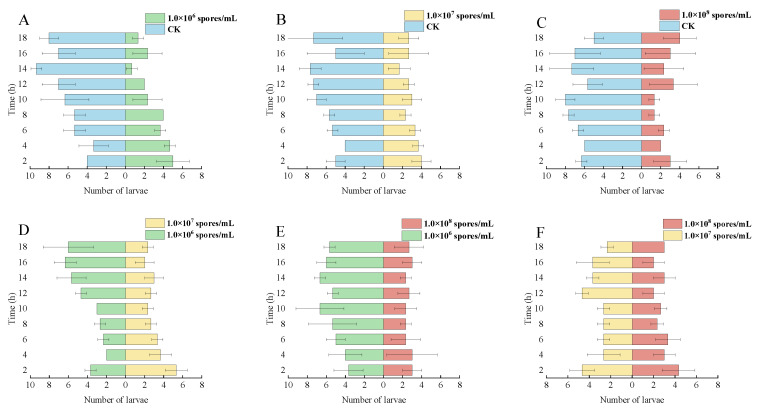
Feeding preference of third instar *P. aenescens* larvae in response to *B. bassiana* treatment: (**A**) 1.0 × 10^6^ spores/mL versus the control group; (**B**) 1.0 × 10^7^ spores/mL versus the control group; (**C**) 1.0 × 10^8^ spores/mL versus the control group; (**D**) 1.0 × 10^6^ spores/mL versus 1.0 × 10^7^ spores/mL; (**E**) 1.0 × 10^6^ spores/mL versus 1.0 × 10^8^ spores/mL; (**F**) 1.0 × 10^7^ spores/mL versus 1.0 × 10^8^ spores/mL.

These results demonstrate that *P. aenescens* exhibits significant avoidance behavior toward *B. bassiana*, which was positively correlated with spore concentration (Figure 3).

### 3.3. Impact of B. bassiana Infection on Growth and Development of P. aenescens

**Figure 4:**
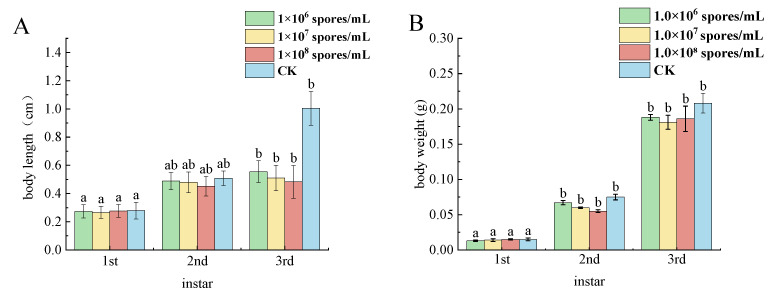
Dynamic changes in body length and weight of *P. aenescens* larvae under *B. bassiana* stress: (**A**) Effect of *B. bassiana* infection on the body length of *P. aenescens* larvae; (**B**) Effect of *B. bassiana* infection on the body weight of *P. aenescens* larvae. Different lowercase letters differ significantly (Duncan’s multiple range test, *p* < 0.05).

*B. bassiana* significantly affected larval growth and development, with higher concentrations resulting in slower growth rates (Table 2 and Table 3; Figure 4). Both body length and weight exhibited significant, dose-dependent reductions relative to controls.

At the end of the first instar, neither body length nor weight differed significantly between treatments and the control. By the end of the second instar, significant growth inhibition was evident: body length was reduced in the 1.0 × 10^8^ spores/mL treatment relative to the control, whereas body weight showed dose-dependent reductions across all treatments. At the end of the third instar, inter-treatment differences in body length were non-significant, yet all treatments remained significantly below control levels; body weight continued to exhibit significant, concentration-dependent reductions (Figure 4).

### 3.4. Physiological Response of P. aenescens to B. bassiana Infection

*Hemocyte dynamics*. To investigate the physiological impact of *B. bassiana* on host immunity, total hemocyte counts were monitored in third-instar larvae following fungal challenge (Table 4). *B. bassiana* infection induced significant, concentration-dependent alterations in circulating hemocyte populations.

At 1.0 × 10^6^ spores/mL, hemocyte counts exhibited a biphasic response: an initial decline to 2.30 × 10^6^ cells by 36 h, followed by a modest recovery phase. At 1.0 × 10^7^ spores/mL, counts decreased to 2.21 × 10^6^ cells by 24 h, followed by a transient rebound to 2.45 × 10^6^ cells at 24–36 h, and then declined significantly to 1.94 × 10^6^ cells thereafter. At 1.0 × 10^8^ spores/mL, the most rapid suppression was observed, with counts falling to 1.86 × 10^6^ cells by 12 h, exhibiting a transient increase to 2.05 × 10^6^ cells at 12–24 h, and a subsequent precipitous decline to 1.34 × 10^6^ cells after 24 h.

Notably, all treatments maintained significantly lower hemocyte counts than the controls throughout the observation period, indicating the sustained immunosuppressive effects of the fungal infection.

All treatment groups differed significantly from the control at all time points, with values consistently lower than the control.

*Melanization responses.* Dissection revealed that successful *B. bassiana* infection induced melanotic nodule formation across all developmental stages (Table 5). Nodule density was closely related to spore concentration, infection duration, and developmental stage. All treatments exhibited significantly elevated nodule counts compared to controls (0 nodules/individual), with pronounced dose-dependent and stage-specific variation.

First-instar larvae displayed progressive accumulation, with counts at 1.0 × 10^8^ spores/mL increasing from 4.67 ± 2.08 (6 h) to 6.33 ± 0.58 (12 h) and 5.33 ± 2.52 (24 h), then rising sharply to 15 ± 2 (36 h) and 17.33 ± 0.58 (48 h). At 1.0 × 10^6^ spores/mL, counts remained lower but reached 12 ± 1 by 48 h. Second-instar larvae exhibited a rapid response, peaking at 17.67 ± 2.08 nodules/individual (24 h, 1.0 × 10^8^ spores/mL), and then declining to 14 ± 1 (36 h) and 13.67 ± 2.08 (48 h). Lower concentrations showed attenuated peaks (11.33 ± 1.53 at 1.0 × 10^7^; 5.33 ± 0.58 at 1.0 × 10^6^). Third-instar larvae showed a delayed but intensified melanization reaction: at 1.0 × 10^8^ spores/mL, counts peaked at 14 ± 1 (24 h), declined to 7.67 ± 1.55 (36 h), and then rebounded to 15.33 ± 2.52 (48 h). Notably, the 1.0 × 10^7^ spores/mL yielded the highest overall count (20.67 ± 2.08 at 48 h).

**Figure 5:**
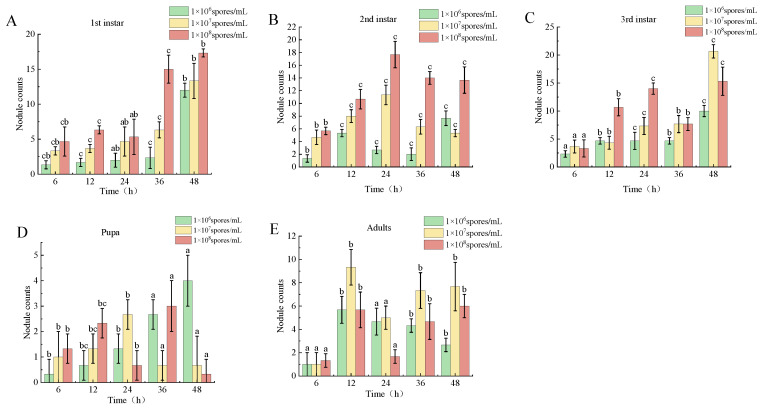
Time course of melanized nodulation in *B. bassiana*-infected *P. aenescens* at various developmental stages: (**A**–**C**) 1st to 3rd instar larvae; (**D**) Pupae; (**E**) adults. Different lowercase letters differ significantly (Duncan’s multiple range test, *p* < 0.05).

Pupae demonstrated the weakest and most variable response, with counts consistently below 4 ± 1 even at the highest concentration, confirming the presence of cuticular or physiological barriers to melanization. Adults manifested an intermediate intensity, peaking at 9.33 ± 1.53 nodules/individual (12 h, 1.0 × 10^7^ spores/mL) (Figure 5).

An integrated analysis revealed that the younger larvae exhibited accelerated responsiveness and elevated encapsulation magnitudes, indicative of a heightened immunological sensitivity to *B. bassiana*. These melanization patterns, convergent with the virulence bioassay data, establish that the optimal intervention window targets early-larval instars (1st–2nd), whereas the pupal stages represent the most recalcitrant phase for biocontrol implementation.

## 4. Discussion

This study examined the entomopathogenic fungus *B. bassiana* as a prospective biocontrol agent against the elm leaf beetle, *P. aenescens*. Laboratory virulence bioassays confirmed pronounced pathogenicity, with mortality rates, mycosis incidence, and lethal velocity exhibiting significant positive correlations with the fungal inoculum concentration. Our LT_50_ values (3.470–19.110 days) are comparable to those reported for *Gonocephalum mongolicum* (6.74 days) [16] and *Monochamus alternatus* (7.77–9.69 days) [14], but the extended pupal LT_50_ (>19 days) exceeds most coleopteran records, suggesting stronger stage-specific defenses in *P. aenescens.* As virulence constitutes a primary determinant of entomopathogenic fungal efficacy, *B. bassiana* demonstrates substantial potential for the integrated management of *P. aenescens* populations [34,35].

Stage-specific variation in pathogenicity was evident, with advanced larval instars displaying heightened resistance—a pattern presumably attributable to cuticular thickening and the protective effects of ecdysis. Consequently, early larval stages (1st–2nd instar) represent the optimal intervention window for fungal application [36,37,38]. In contrast, the pupal stages presented the most formidable challenge for biocontrol, likely reflecting the defensive properties of the pupa and the metabolically quiescent state characteristic of this developmental phase [39,40]. Similarly, the egg stage exhibited strong resistance to *B. bassiana* infection, likely attributable to the highly sclerotized, waterproof chorion that physically impedes fungal penetration, the absence of cuticular hydrocarbons required for fungal attachment, and the shielding effect of compact egg masses wherein inner eggs are protected by the outer layer [41,42,43].

Dual-choice feeding assays revealed that *P. aenescens* consistently avoided *B. bassiana*-treated leaves, exhibiting pronounced antifeedant behavior [44,45]. This concentration-dependent avoidance aligns with observations in *Ootheca mutabilis* (>10^7^ spores/mL) [19] but contrasts with *Drosophila melanogaster*, where stimulated feeding was reported at similar doses [23], suggesting metabolite-specific phagostimulatory thresholds across host taxa. Because low concentrations (1.0 × 10^6^ spores/mL) did not induce strong avoidance while maintaining high pathogenicity, and maintaining infected individuals facilitates pathogen spread, this concentration may be suitable for further evaluation under field application [46]. However, we emphasize that this recommendation is preliminary and based solely on laboratory dipping bioassays. Foliar spraying in the field involves markedly different environmental variables (UV exposure, temperature fluctuations, rainfall, leaf surface chemistry) that may significantly affect conidial viability and efficacy. Field trials are therefore essential to validate these laboratory findings before practical application.

Mechanism of root-effects. The root-drenching method was designed to evaluate systemic, endophyte-mediated effects rather than direct contact toxicity. The verification of *B. bassiana* colonization in the leaf tissues confirms that the observed lethal and sublethal effects on the larvae were mediated through endophytic transmission rather than direct exposure to fungal propagules on the leaf surface. The growth inhibition observed (20–26% weight reduction in second instar) is comparable to the endophytic *B. bassiana* effects reported on stem-borers [21], but weaker than that induced by direct topical exposure, suggesting metabolite-mediated rather than propagule-driven effects. This distinguishes our approach from the topical bioassays (Section 2.2) and provides evidence for the plant-mediated biocontrol potential. However, we acknowledge that the relative contributions of endophytic metabolites versus viable fungal propagules within the tissues remain to be elucidated.

Hemocyte counts in the infected larvae were significantly lower than those in the controls, showing a pattern of an initial decrease, a transient increase, and a subsequent decrease. The hemocyte depletion we observed (to 1.34 × 10^6^ cells at 48 h) is more severe than that reported for *Bombyx mori* (2.09 × 10^6^) [24] but less extreme than that observed in *Algedonia coclesalis* infected with *Metarhizium anisopliae* [25], suggesting host-specific immune suppression intensity. This suggests that *B. bassiana* affects hemocytes and immune function [47,48], given that hemocytes are crucial immune components [49]. Initial decreases likely result from plasmatocyte lysis, while the subsequent increase reflects prohemocyte differentiation and the new plasmatocyte production [50].

Melanization responses were weakest in pupae and strongest in young larvae, following an increase–decrease–increase pattern. The triphasic pattern in second-instar larvae differs from progressive accumulation in *B. mori* [24] and monophasic responses in *Galleria mellonella* [27], indicating age-dependent immune maturation in *P. aenescens*. This indicates rapid immune defense activation [51]. *B. bassiana* disrupts cellular immunity and key enzymes, weakening defense and increasing nodule formation [52]. However, excessive melanization may cause self-damage, explaining subsequent decreases [53]. The paradox that stronger melanization coincides with higher mortality in early instars challenges the assumption that robust immune activation guarantees survival [28,29].

This study provides preliminary evidence on virulence and physiological effects. Several limitations warrant acknowledgment: the dipping method delivers uniform coverage unlike field deposition; the leaf-disk assay measures settling rather than consumption; immune assays were restricted to third instar; and laboratory conditions (25 °C, 75% RH) optimize germination but rarely occur in field environments where UV and desiccation reduce viability. Future research should employ transcriptomic and metabolomic analyses to elucidate mechanisms [54,55], evaluate formulation and application methods for improving fungal persistence under field-relevant conditions, and investigate compatibility with reduced-rate chemical pesticides [56,57,58]. Non-target effects on beneficial insects should also be assessed.

## 5. Conclusions

In conclusion, *B. bassiana* SYAUBB001 exhibits high pathogenicity against *P. aenescens*, with first-instar larvae showing the highest susceptibility (corrected mortality: 73.33%; LT_50_: 3.470 days) and pupae/eggs the lowest (corrected mortality: 2.2–57.8%; LT_50_: 5.158–19.110 days). Adults exhibited delayed mortality but 100% mycosis by day 7. Early-instar larvae represent the optimal intervention window.

Infected larvae displayed concentration-dependent avoidance of treated foliage, significantly reduced body length and weight gain, decreased total hemocyte counts, and enhanced melanization and nodulation responses—more pronounced in younger larvae.

*B. bassiana* acts through direct mortality and sublethal pathways (immune suppression, energy-costly defense responses, feeding/growth disruption). The coincident high mortality and strong immune activation in early instars suggest that immune responses alone are insufficient to overcome infection.

Based on these findings, we recommend targeting first- and second-instar larvae with *B. bassiana* applications at concentrations of 1.0 × 10^7^–1.0 × 10^8^ spores/mL for effective biocontrol under laboratory conditions. The concentration of 1.0 × 10^6^ spores/mL may warrant further evaluation for scenarios where antifeedant effects are undesirable yet pathogen establishment is desired. These recommendations are derived from controlled laboratory bioassays and require validation through field trials before practical implementation.

## Figures and Tables

**Table 1:** Laboratory virulence of *B. bassiana* against different developmental stages of *P. aenescens*.

Stage	Concentration (Spores/mL)	Corrected Mortality (%)	Mycosis Rate (%)	Regression Equation	R2	LT_50_ (d)	95% CI
Egg	1.0 × 10^6^	28.87	44.47	y = −4.10x + 52.50	0.9497	7.092	6.179–8.610
	1.0 × 10^7^	37.80	55.53	y = −4.35x + 52.08	0.9352	6.581	5.714–7.853
	1.0 × 10^8^	57.80	73.33	y = −5.35x + 51.75	0.8753	5.158	4.456–5.894
1st instar	1.0 × 10^6^	40.00	77.80	y = −8.40x + 59.40	0.8412	5.399	4.893–5.884
	1.0 × 10^7^	48.87	68.87	y = −7.20x + 52.20	0.9796	5.006	4.397–5.602
	1.0 × 10^8^	73.33	100.00	y = −8.91x + 45.20	0.8337	3.470	2.986–3.911
2nd instar	1.0 × 10^6^	15.53	73.33	y = −9.26x + 62.40	0.8679	5.998	5.549–6.431
	1.0 × 10^7^	20.00	93.33	y = −9.69x + 61.40	0.9363	5.480	5.059–5.877
	1.0 × 10^8^	33.33	95.53	y = −10.46x + 60.60	0.9492	4.396	3.922–4.834
3rd instar	1.0 × 10^6^	6.67	44.47	y = −8.49x + 61.20	0.8260	6.953	6.459–7.477
	1.0 × 10^7^	6.67	88.87	y = −10.46x + 63.60	0.8786	5.966	5.525–6.387
	1.0 × 10^8^	11.13	97.13	y = −10.37x + 63.80	0.8757	6.028	5.584–6.452
Pupa	1.0 × 10^6^	0.00	2.20	y = −0.75x + 47.42	0.4561	19.110	―
	1.0 × 10^7^	2.20	8.87	y = −1.40x + 49.33	0.5709	13.603	―
	1.0 × 10^8^	4.47	40.00	y = −5.83x + 57.40	0.7078	7.961	7.395–8.812
Adult	1.0 × 10^6^	44.47	100.00	y = −7.35x + 61.75	0.8415	5.392	4.853–5.890
	1.0 × 10^7^	46.67	100.00	y = −7.35x + 61.75	0.8415	5.392	4.853–5.890
	1.0 × 10^8^	53.33	100.00	y = −7.35x + 59.75	0.8752	5.018	4.491–5.498

**Table 2:** Body length of *P. aenescens* larvae at the end of each instar under *B. bassiana* stress (cm, mean ± SD).

Instar	Control	1 × 10^6^ Spores/mL	1 × 10^7^ Spores/mL	1 × 10^8^ Spores/mL
1st	0.279 ± 0.060 ^a^	0.273 ± 0.047 ^a^	0.267 ± 0.043 ^a^	0.277 ± 0.045 ^a^
2nd	0.508 ± 0.052 ^a^	0.488 ± 0.61 ^ab^	0.479 ± 0.074 ^ab^	0.452 ± 0.070 ^b^
3rd	1.004 ± 0.120 ^a^	0.555 ± 0.077 ^b^	0.510 ± 0.091 ^b^	0.483 ± 0.116 ^b^

Note: Values within rows followed by different lowercase letters differ significantly (Duncan’s multiple range test, *p* < 0.05).

**Table 3:** Body weight of *P. aenescens* larvae at the end of each instar under *B. bassiana* stress (g, mean ± SD).

Instar	Control	1 × 10^6^ Spores/mL	1 × 10^7^ Spores/mL	1 × 10^8^ Spores/mL
1st	0.015 ± 0.002 ^a^	0.013 ± 0.001 ^a^	0.014 ± 0.002 ^a^	0.015 ± 0.001 ^a^
2nd	0.075 ± 0.004 ^a^	0.067 ± 0.003 ^b^	0.060 ± 0.001 ^c^	0.055 ± 0.002 ^d^
3rd	0.208 ± 0.014 ^a^	0.188 ± 0.004 ^b^	0.181 ± 0.010 ^bc^	0.186 ± 0.018 ^c^

Note: Values within rows followed by different lowercase letters differ significantly (Duncan’s multiple range test, *p* < 0.05).

**Table 4:** Total hemocyte count in third instar *P. aenescens* larvae after *B. bassiana* infection (10^6^ cells, mean ± SD).

Time (h)	1 × 10^6^ Spores/mL	1 × 10^7^ Spores/mL	1 × 10^8^ Spores/mL	Control
6	3.36 ± 0.04 ^Ac^	3.44 ± 0.08 ^Ab^	3.35 ± 0.03 ^Ac^	3.88 ± 0.01 ^Aa^
12	2.62 ± 0.11 ^Bb^	2.36 ± 0.08 ^Bc^	1.86 ± 0.10 ^Cd^	3.85 ± 0.01 ^Aa^
24	2.30 ± 0.06 ^Db^	2.21 ± 0.09 ^Cb^	2.05 ± 0.10 ^Bc^	3.87 ± 0.02 ^Aa^
36	2.45 ± 0.09 ^Cb^	2.45 ± 0.02 ^Bb^	1.89 ± 0.14 ^BCc^	3.86 ± 0.02 ^Aa^
48	2.55 ± 0.11 ^BCb^	1.94 ± 0.02 ^Dc^	1.34 ± 0.10 ^Dd^	3.86 ± 0.02 ^Aa^

Note: Different lowercase letters within rows indicate significant differences between treatment groups and the control at each time point; different uppercase letters within columns indicate significant differences among time points for the same treatment (Duncan’s new multiple range test, *p* < 0.05).

**Table 5:** Melanotic nodule formation in *P. aenescens* at various developmental stages after *B. bassiana* infection (nodules/individual, mean ± SD).

Stage	Concentration	6 h	12 h	24 h	36 h	48 h
1st instar	1 × 10^6^	1.33 ± 0.58 ^cb^	1.67 ± 0.58 ^c^	2.00 ± 1.00 ^ab^	2.33 ± 1.53 ^c^	12.00 ± 1.00 ^b^
	1 × 10^7^	3.33 ± 0.58 ^ab^	3.67 ± 0.58 ^b^	4.67 ± 2.08 ^a^	6.33 ± 1.15 ^b^	13.33 ± 2.52 ^b^
	1 × 10^8^	4.67 ± 2.08 ^a^	6.33 ± 0.58 ^a^	5.33 ± 2.52 ^a^	15.00 ± 2.00 ^a^	17.33 ± 0.58 ^a^
	Control	0	0	0	0	0
2nd instar	1 × 10^6^	1.33 ± 0.58 ^b^	5.33 ± 0.58 ^c^	2.67 ± 0.58 ^c^	2.00 ± 1.00 ^c^	7.67 ± 1.15 ^c^
	1 × 10^7^	4.67 ± 1.15 ^a^	8.00 ± 1.00 ^b^	11.33 ± 1.53 ^b^	6.33 ± 1.15 ^b^	5.33 ± 0.58 ^b^
	1 × 10^8^	5.67 ± 0.58 ^a^	10.67 ± 1.53 ^a^	17.67 ± 2.08 ^a^	14.00 ± 1.00 ^a^	13.67 ± 2.08 ^a^
	Control	0	0	0	0	0
3rd instar	1 × 10^6^	2.33 ± 0.58 ^a^	4.67 ± 0.58 ^b^	4.67 ± 1.53 ^c^	4.67 ± 0.58 ^b^	10.00 ± 1.00 ^c^
	1 × 10^7^	3.67 ± 1.15 ^a^	4.33 ± 1.15 ^b^	7.33 ± 1.53 ^b^	7.67 ± 1.53 ^a^	20.67 ± 2.08 ^a^
	1 × 10^8^	3.33 ± 1.53 ^a^	10.67 ± 1.53 ^a^	14.00 ± 1.00 ^a^	7.67 ± 1.55 ^a^	15.33 ± 2.52 ^b^
	Control	0	0	0	0	0
Pupa	1 × 10^6^	0.33 ± 0.58 ^a^	0.67 ± 0.58 ^bc^	1.33 ± 0.58 ^b^	2.67 ± 0.58 ^a^	4.00 ± 1.00 ^a^
	1 × 10^7^	1.00 ± 1.00 ^ab^	1.33 ± 0.58 ^b^	2.67 ± 0.58 ^a^	0.67 ± 0.58 ^b^	0.67 ± 1.15 ^b^
	1 × 10^8^	1.33 ± 0.58 ^a^	2.33 ± 0.58 ^a^	0.67 ± 0.58 ^bc^	3.00 ± 1.00 ^a^	0.33 ± 0.58 ^b^
	Control	0	0	0	0	0
Adult	1 × 10^6^	1.00 ± 1.00 ^a^	5.67 ± 1.15 ^b^	4.67 ± 1.15 ^a^	4.33 ± 0.58 ^b^	2.67 ± 0.58 ^b^
	1 × 10^7^	1.00 ± 1.00 ^a^	9.33 ± 1.53 ^a^	5.00 ± 1.00 a	7.33 ± 1.53 ^a^	7.67 ± 2.08 ^a^
	1 × 10^8^	1.33 ± 0.58 ^a^	5.67 ± 1.53 ^b^	1.67 ± 0.58 ^b^	4.67 ± 1.53 ^b^	6.00 ± 1.00 ^a^
	Control	0	0	0	0	0

Note: Values within rows followed by different lowercase letters differ significantly from control at each time point (Duncan’s multiple range test, *p* < 0.05).

## Data Availability

The original contributions presented in this study are included in the Article. Further inquiries can be directed to the corresponding author.
